# Transfusion Tsunami: A 132-Liter Resuscitation Using Crystalloids, Colloids, Blood, and Coagulation Factors During Liver Transplantation

**DOI:** 10.7759/cureus.88266

**Published:** 2025-07-18

**Authors:** Laurence Weinberg, Peter Le, Vidhura Ratnasekara, Nattaya Raykageeraroj, Je Min A Suh, Dong-Kyu Lee

**Affiliations:** 1 Department of Anesthesia, Austin Health, Melbourne, AUS; 2 Department of Critical Care, University of Melbourne, Melbourne, AUS; 3 Department of Anesthesiology, Faculty of Medicine, Siriraj Hospital, Mahidol University, Bangkok, THA; 4 Department of Anesthesiology and Pain Medicine, Dongguk University Ilsan Hospital, Goyang, PRK

**Keywords:** clinical anaesthesiology, coagulation case reporting, cross match to transfusion ratio, hemodynamic monitoring, liver transplant complications, liver transplant physician, liver transplant surgery, massive transfusion protocol, red blood cell transfusion, transfusion practices

## Abstract

Despite advancements in surgical technique and anesthetic management, liver transplantation continues to pose a significant risk of intraoperative bleeding requiring substantial transfusion support. We describe an adult patient who underwent an orthotopic liver transplant and received a total of 132 liters of fluid within a 24-hour period, comprising 50 packed red blood cell units, 20 pooled platelet units, 24 fresh frozen plasma units, and 100 cryoprecipitate units. This case emphasizes the multifaceted approach to ultra-massive transfusion in patients with advanced liver disease who undergo liver transplantation. The immediate postoperative course was complicated by primary graft failure requiring re-transplantation. Although the postoperative course was complicated by sepsis, respiratory failure, bile leak, portal vein thrombosis, and renal impairment, with ongoing medical management and rehabilitation, the patient made a full recovery. One year post-transplant, graft function remains normal, and the patient has returned to his previous activities of daily living.

## Introduction

Despite advancements in surgical technique and anesthetic management, liver transplantation (LT) continues to pose a significant risk of intraoperative bleeding requiring substantial transfusion support [1]. Approximately 5.8% of patients undergoing LT receive ultra-massive transfusion (UMT), typically defined as more than 20 units of packed red blood cells (PRBC) [2]. UMT during LT is associated with increased morbidity, mortality, and healthcare costs. End-stage liver disease (ESLD) results in complex alterations of the coagulation system, impacting both procoagulant and anticoagulant factors. This leads to a ‘rebalanced’ but unstable homeostasis that predisposes patients to either excessive bleeding or thrombotic events [3].

While fixed-ratio transfusion protocols, commonly used in trauma, have been proposed for managing massive bleeding, their use in LT remains controversial due to the dynamic and unpredictable nature of coagulopathy in ESLD. Research shows that relying solely on standard coagulation testing and fixed-ratio transfusion protocols during LT can be suboptimal [4]. Therefore, viscoelastic testing (VET) has increasingly been used to guide the management of intraoperative bleeding and transfusion of blood products in real-time, allowing for a more individualized, goal-directed approach [5].

To our knowledge, intraoperative resuscitation exceeding 120 liters has not been reported in the context of LT. We report a case of a 62-year-old male with ESLD who received a total of 132 liters of fluid resuscitation - including crystalloids, colloids, blood products, and coagulation factors - during LT, guided by VET. This case emphasizes the extreme complexity of UMT management and supports the value of personalized, VET-guided transfusion strategies in this high-risk setting.

## Case presentation

A 62-year-old male (92 kg) with a history of metabolic dysfunction-associated steatotic liver disease (MASLD)-related cirrhosis underwent orthotopic LT. His chronic liver disease was complicated by portal hypertension, esophageal varices, refractory ascites, and spontaneous bacterial peritonitis. Cardiovascular risk factors included type 2 diabetes mellitus (T2DM), primary hypogonadism, and a prior coronary artery bypass graft (CABG) surgery for ischemic heart disease five years earlier. He resided independently at home and maintained full autonomy in activities of daily living (ADLs). At admission, his medication regimen comprised intravenous terlipressin (3.4 mg/24 hr via continuous home-based infusion), rifaximin (550 mg orally twice daily), and trimethoprim-sulfamethoxazole (160-800 mg orally once daily).

Investigations

His pre-transplant hematology and coagulation profiles were relatively normal: hemoglobin 109 g/L, platelets 105 × 10⁹/L, activated partial thromboplastin time (aPTT) 32 seconds, fibrinogen 2.9 g/L, and international normalized ratio (INR) 1.3. His urea and electrolytes were unremarkable (creatinine 62 µmol/L, urea 4.9 mmol/L, bicarbonate 26 mmol/L). Liver function test results were as follows: bilirubin 31 µmol/L, alanine transaminase (ALT) 37 units/L, aspartate transaminase (AST) 85 units/L, and albumin 26 g/L. His Model for End-Stage Liver Disease (MELD) score was 17.

The patient underwent preoperative dobutamine stress echocardiography (DSE), which demonstrated no evidence of inducible ischemia, as evidenced by the absence of new or worsening wall motion abnormalities. Resting echocardiographic evaluation revealed normal left ventricular chamber dimensions and preserved systolic function, with an ejection fraction of 67%. Additionally, grade 1 diastolic dysfunction was observed, consistent with impaired relaxation. Right ventricular size and systolic function were within normal limits, as confirmed by a tricuspid annular plane systolic excursion (TAPSE) of 23 mm. Non-invasive estimation of pulmonary artery pressures yielded values of 26/12 mmHg (systolic/diastolic), with a derived mean pulmonary artery pressure of 17 mmHg.

**Figure 1:**
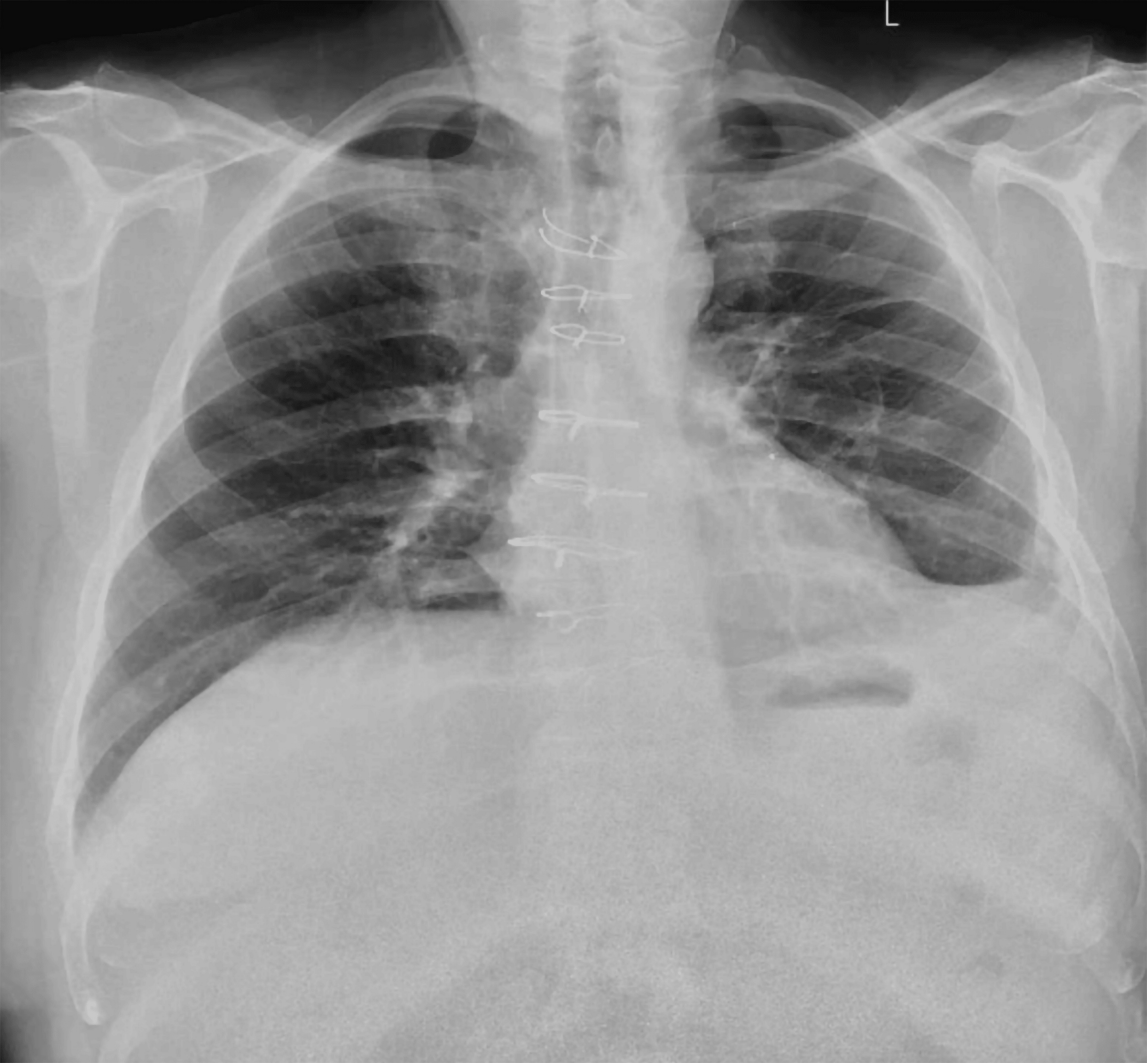
Pre-operative chest xray

**Figure 2:**
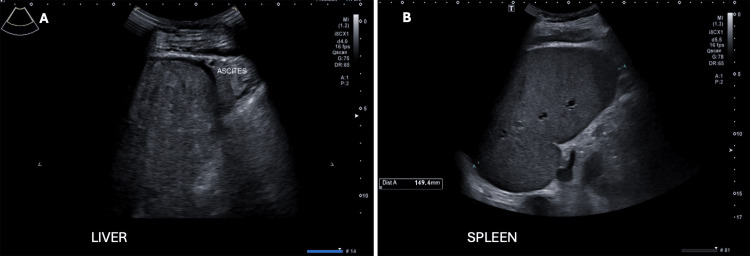
Pre-operative abdominal ultrasound with liver demonstrating a coarsened echotexture and nodular surface contour with small volume ascites, consistent with cirrhosis (A), and splenomegaly (B)

Chest radiography revealed a small left pleural effusion, a right-sided peripherally inserted central catheter (PICC), and evidence of previous cardiac surgery (Figure 1). Respiratory function tests were normal. Abdominal ultrasound demonstrated a coarsened echotexture and nodular contour of the liver, consistent with cirrhosis. The main portal vein was of normal diameter and demonstrated normal bidirectional flow with no evidence of thrombosis. Splenomegaly and small-volume ascites were evident, consistent with portal hypertension (Figure 2).

Treatment

**Figure 3:**
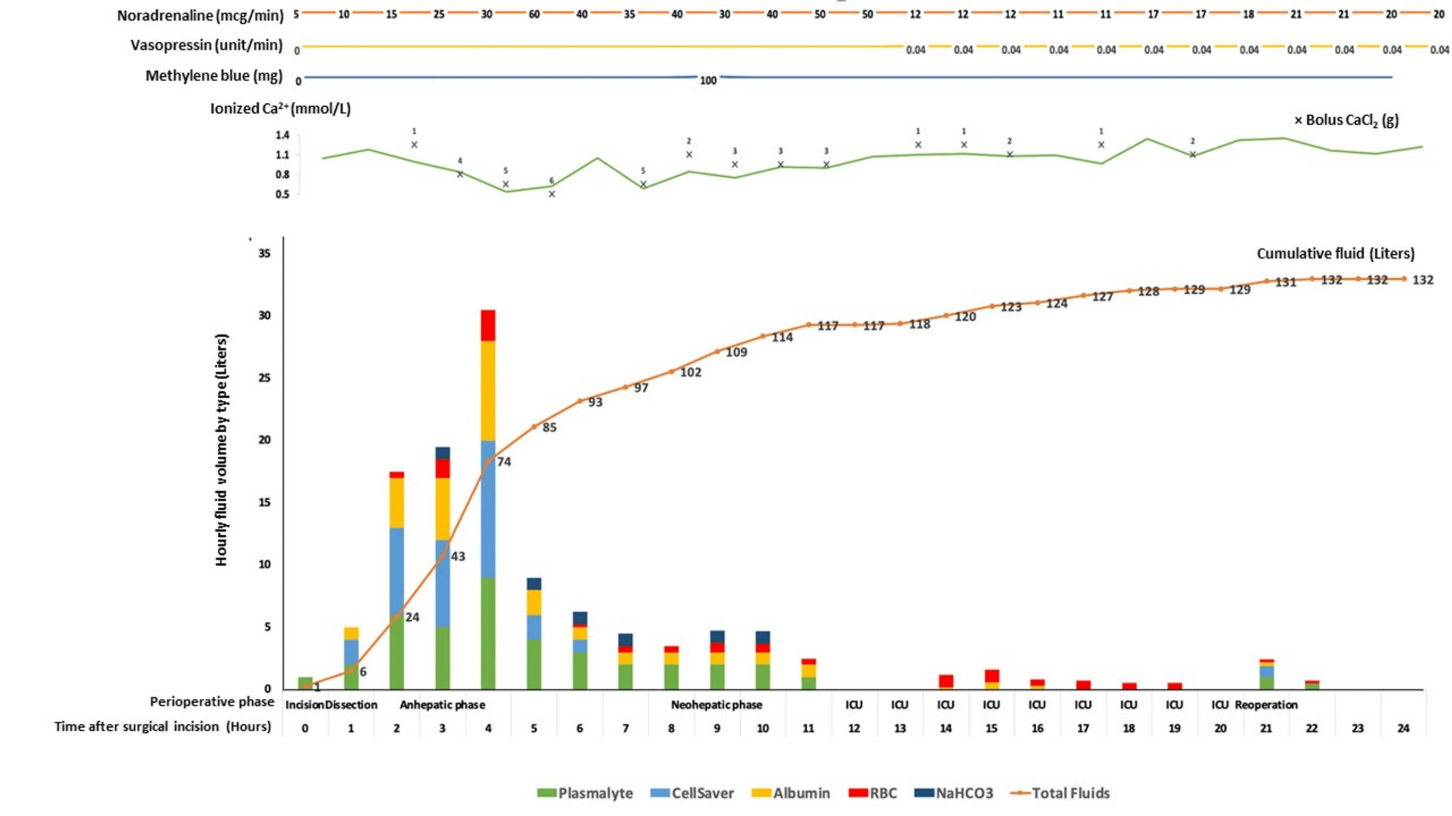
Cumulative and hourly intraoperative and postoperative fluid resuscitation, vasoactive medication use, and calcium correction over 24 hours following liver transplantation The bar chart (bottom panel) displays hourly fluid volume stratified by type (Plasmalyte, cell saver autologous blood, albumin, packed red blood cells, sodium bicarbonate), with the orange line indicating total cumulative fluid administered. Top panels show concurrent vasoactive medication doses (noradrenaline, vasopressin, methylene blue) and ionized calcium trends, with calcium chloride boluses marked by '×' and labeled in grams. Time (X-axis) is shown in hours from surgical incision, grouped by surgical phases (dissection, anhepatic, neohepatic) and intensive care unit (ICU) period.

Intraoperatively, dense adhesions around the liver resulting from a previous cholecystectomy were encountered during the pre-anhepatic phase of his LT, resulting in significant hemorrhage during adhesiolysis. Initially, the patient was reinfused with autologous blood via a cell saver system, alongside additional transfusions of albumin and crystalloid solutions. Figure 3 graphically depicts the rapid accumulation of fluid volumes during the dissection and anhepatic phases, with escalating requirements for crystalloids, albumin, and blood products paralleling increasing hemodynamic instability and ongoing hemorrhage. Concurrently, vasopressor use intensified, reflecting worsening vasoplegia and hemodynamic compromise.

**Figure 4:**
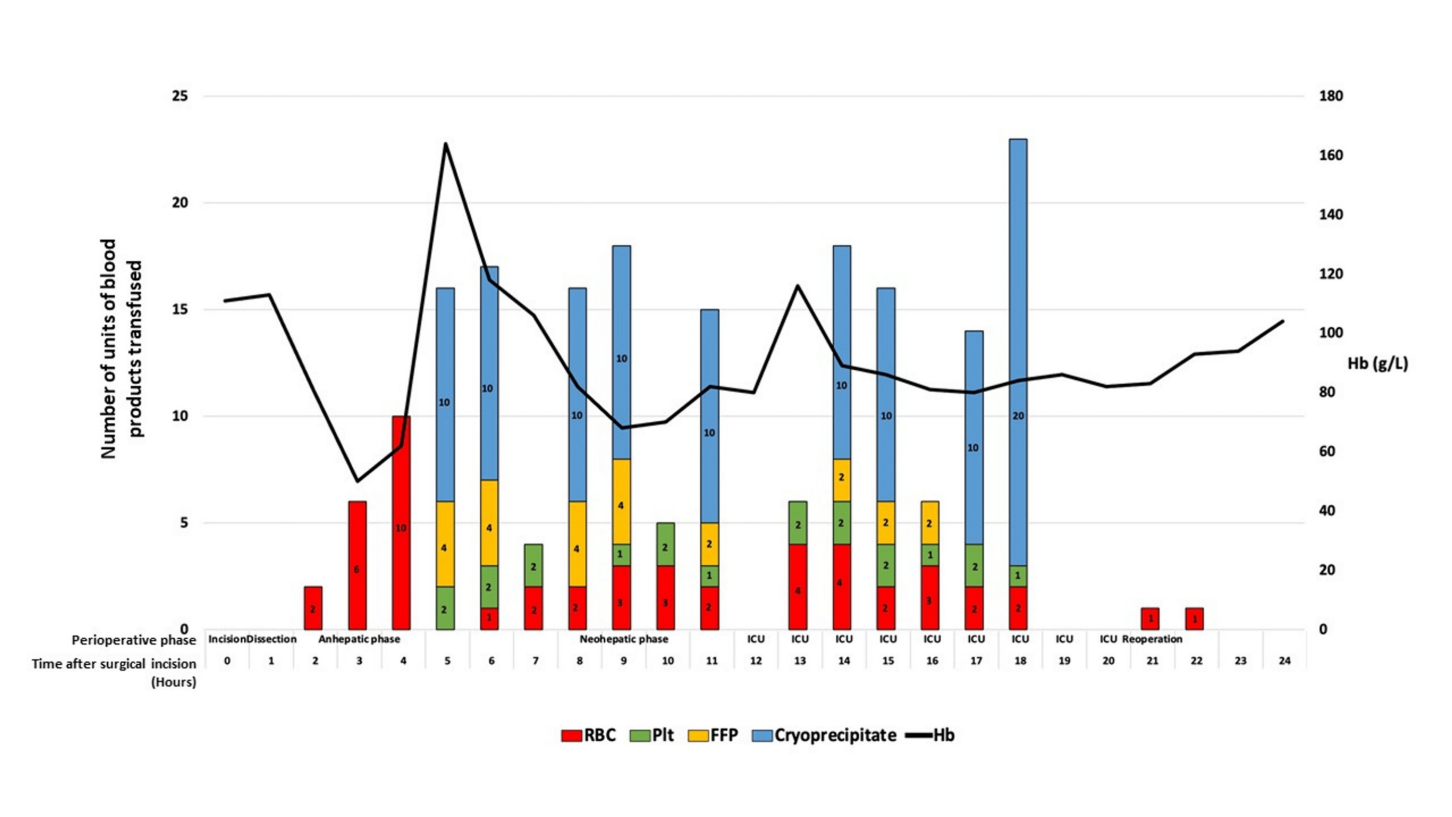
Intraoperative and postoperative blood product transfusion and hemoglobin trends over 24 hours following liver transplantation. This figure illustrates the hourly volume and types of blood products transfused (red blood cells [RBC], platelets [Plt], fresh frozen plasma [FFP], and cryoprecipitate) during and after liver transplantation surgery over a 24-hour period post-surgical incision. The stacked bar chart shows the number of units transfused each hour, categorized by blood product type with color coding (RBC in red, Plt in green, FFP in yellow, and cryoprecipitate in blue). Numbers within each bar segment indicate units transfused. The black line represents the concurrent hemoglobin (Hb) levels (g/L) measured throughout the same timeframe, plotted on the secondary Y-axis on the right. Perioperative phases, including incision, dissection, anhepatic phase, neohepatic phase, intensive care unit (ICU) stay, and reoperation, are labeled along the X-axis. This visualization captures transfusion patterns and hemoglobin fluctuations in the critical perioperative period of liver transplantation.

Despite these measures, his hemoglobin levels began to decline rapidly during the dissection phase, while vasopressor requirements increased, necessitating activation of massive transfusion protocols. Figure 4 illustrates the detailed breakdown of blood components transfused over time, revealing the prioritization of PRBCs and cryoprecipitate during periods of peak hemorrhage. The concurrent downward trend in hemoglobin concentration (black line) underscores the severity of ongoing blood loss despite aggressive replacement therapy.

Surgical bleeding from adhesiolysis sites persisted during the anhepatic phase, resulting in precipitous drops in hemoglobin to 50 g/L, despite prioritized transfusion of PRBCs and autologous blood from cell salvage (Figure 4). Concurrently, the patient experienced reductions in temperature, central venous pressure, and ionized calcium levels, which were managed with active patient warming, warmed fluid resuscitation, and calcium chloride infusions.

Upon receipt of the donor liver, caval anastomosis was initiated using a piggyback technique. Subsequently, gross swelling of the small bowel and colon was observed, prompting the discovery of portal vein non-patency. This necessitated dissection of the infracolic superior mesenteric vein and placement of a jump graft using the donor iliac vein. Following this, a donor liver cavocavostomy was performed. The donor hepatic artery was then anastomosed to the recipient hepatic artery.

Bleeding persisted following reperfusion of the new graft, despite achieving a stable hemoglobin concentration exceeding 80 g/L, normothermia, correction of metabolic acidosis with bicarbonate infusion, and administration of tranexamic acid to prevent hyperfibrinolysis. Ultimately, complete hemostasis during the neohepatic phase proved difficult to achieve. As the patient experienced profound hypotension at multiple time points, a damage control strategy was adopted, which involved packing the abdomen with gauze and closing the wound using a negative pressure dressing.

The surgery lasted 11 hours and 27 minutes and required a total transfusion of 31 units of PRBCs, 18 units of fresh frozen plasma (FFP), 10 units of pooled platelets, and 50 units of cryoprecipitate, all guided by VET using thromboelastography (TEG). Longitudinal TEG values across six key intraoperative time points - with postulated mechanisms of bleeding and point-of-care management - are summarized in Table 1. The total intraoperative infusion volume was 103,023 mL, comprising 39,000 mL of PlasmaLyte, 29,315 mL of autologous blood cell salvage, 26,100 mL of albumin, 7,708 mL of PRBCs, and 900 mL of washed donor red blood cells. The cumulative volume of fluids, blood products, and coagulation factors administered is presented graphically in Figure 3.

**Table 1:** Serial thromboelastographic parameters and clinical interpretation across intraoperative phases TEG Parameter Glossary: - R time (Reaction time): Time from test initiation to initial fibrin formation (2 mm amplitude); reflects coagulation factor activity. - K time (Kinetics time): Time from initial fibrin formation to a 20 mm amplitude; reflects the speed of clot strengthening, dependent on fibrinogen and platelet function. - Alpha Angle: Angle between the baseline and the slope of clot formation; reflects the rate of fibrin build-up and platelet–fibrin interaction. - MA (Maximum Amplitude): Represents the maximal clot strength; primarily reflects platelet function and fibrin interaction. - LY30 (Lysis at 30 minutes): Percentage of clot lysis 30 minutes after MA; indicates the degree of fibrinolysis. Normal reference ranges for TEG parameters: R time: 4.6–9.1 minutes; K time: 0.8–2.1 minutes; Alpha Angle: 63–78°; MA: 52.0–69.0 mm; LY30: 0.0–3.2%. Abbreviations: TEG, thromboelastographic; MA, maximum amplitude; ICU, intensive care unit

TEG result	Interpretation in relation with clinical context	Coagulation management
Baseline R time: 5.6 min K time: 1.3 min Alpha Angle: 55.0° MA: 51.4 mm LY 30: 0.9%	Near-normal parameters suggest preserved synthetic liver function. - Mildly decreased MA likely due to portal hypertension and thrombocytopenia.	No coagulation products administered
Dissection phase 1 hour post-incision R time: 14.8 min K time: 4.5 min Alpha Angle: 51.0° MA: 48.0 mm LY 30: 1.1%	Bleeding likely due to portal hypertension, variceal bleeding, thrombocytopenia, and dilutional coagulopathy. - Prolonged R time suggests clotting factor deficiency from hemodilution and consumption. - Prolonged K time and reduced MA indicate platelet dysfunction and dilutional effects.	Start transfusion - Cell saver blood returned - Additional packed red blood cells transfused - No coagulation factors were administered
Anhepatic phase 5 hours post-incision R time: 20.1 min K time: 13.4 min Alpha Angle: 16.5° MA: 38.0 mm LY 30: 14% 7 hours post-incision R time: 21.6 min K time: 99.7 min Alpha Angle: – MA: 11.0 mm LY 30: –	Severe coagulopathy due to dilutional loss and consumption of clotting factors, fibrinogen, and platelets. - Prolonged R and K times with significantly reduced MA suggest worsening coagulation factor deficiency and platelet dysfunction. - Elevated LY30 indicates emerging hyperfibrinolysis. Profound coagulopathy with near-complete clot failure. - Markedly prolonged R and K times reflect extreme depletion of coagulation factors. - Severely reduced MA indicates advanced thrombocytopenia and platelet dysfunction Likely compounded by hypofibrinogenemia and impaired synthetic liver function	Massive transfusion in progress - Cell saver blood returned - Ongoing packed red blood cells transfusion - Coagulation factors replacement with fresh frozen plasma, cryoprecipitate and platelets
Reperfusion phase 60 minutes post-reperfusion R time: 25.2 min K time: 61.5 min Alpha Angle: 21.1° MA: 43.2 mm LY 30: 64%	Severe ongoing bleeding due to coagulopathy from consumption and dilutional loss of coagulation components . - Markedly prolonged R and K times indicate coagulation factor depletion. - Severely reduced MA reflects significant platelet depletion. - Markedly elevated LY30 suggests hyperfibrinolysis. Graft reperfusion may have triggered a heparin-like effect, contributing to further coagulation impairment.	Massive transfusion in progress - Tranexamic acid administrated to correct fibrinolysis - Cell saver blood returned - Ongoing packed red blood cells transfusion - Coagulation factors replacement with fresh frozen plasma, cryoprecipitate and platelets
Closure (ICU admission) R time: 14.7 min K time: 7.1 min Alpha Angle: 27.0° MA: 24.0 mm LY 30: 4.0%	Ongoing bleeding at ICU due to persistent coagulopathy - Prolonged R and K times and markedly reduced MA, consistent with clotting factor deficiency and platelet dysfunction. - Slight improvement in LY30 suggests reduced fibrinolytic activity. - Persistently low MA likely due to residual thrombocytopenia and hypofibrinogenemia.	- No coagulation factors administered - Platelet transfusion in view of ongoing oozing and reduced clot strength (MA)

**Figure 5:**
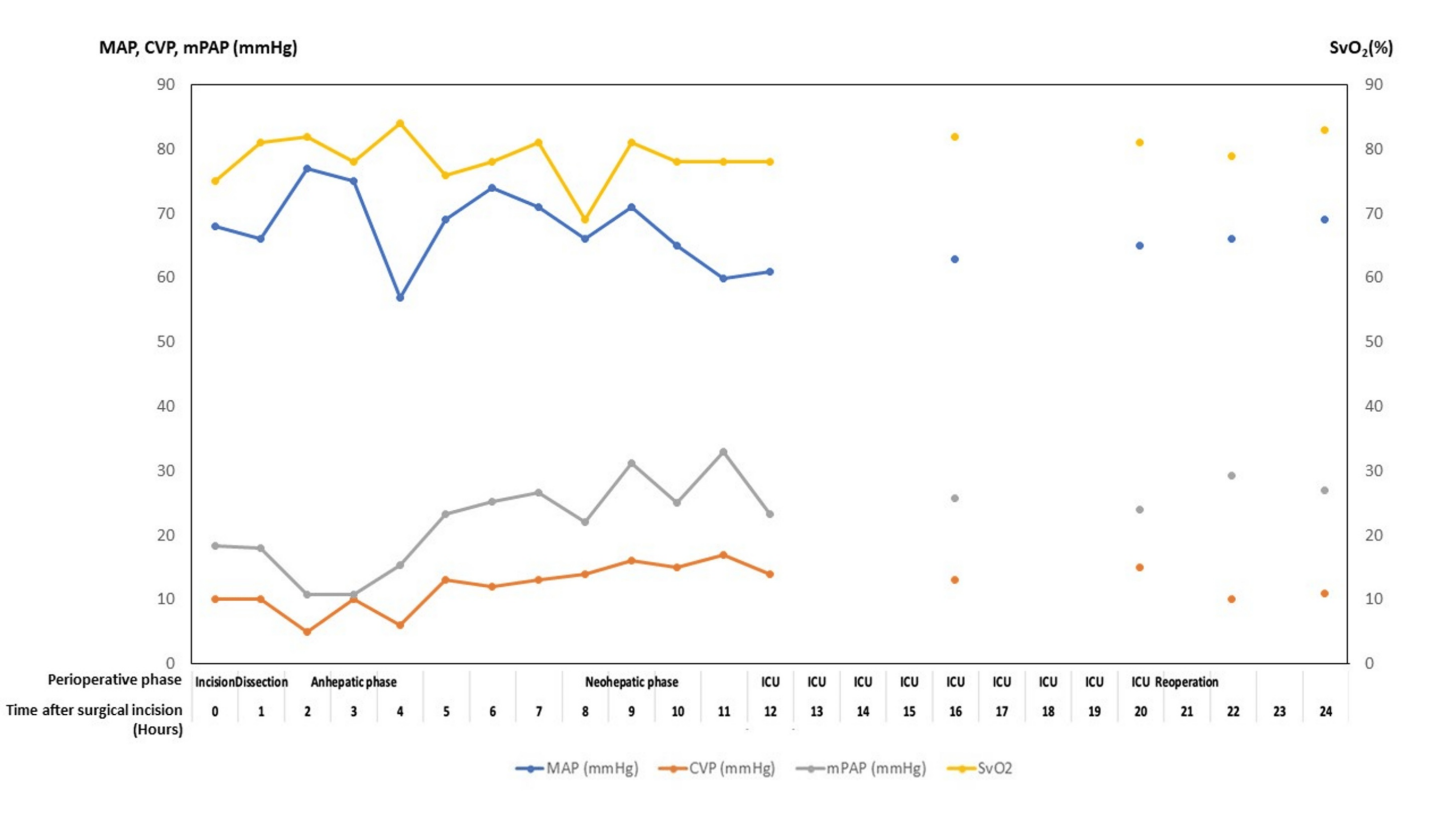
Hemodynamic parameters: MAP, CVP, mPAP, and SvO2 Multi-line graph tracking mean arterial pressure (MAP), central venous pressure (CVP), mean pulmonary artery pressure (mPAP), and mixed venous oxygen saturation (SvO₂, %) over 24 hours post-surgery. These measurements provide insight into cardiovascular function and oxygen delivery during the complex perioperative period.

**Figure 6:**
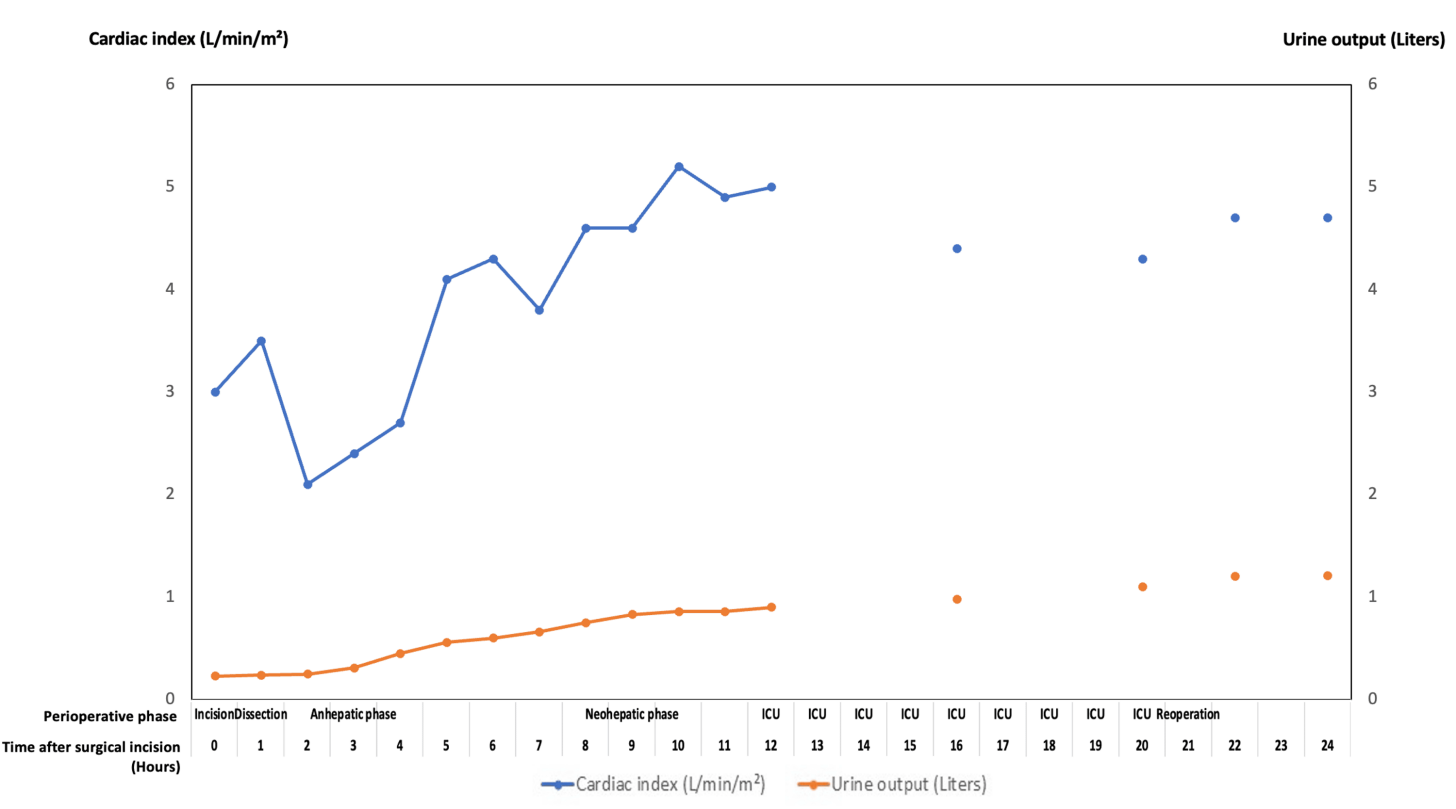
Cardiac index and urine output trend over perioperative and ICU period Dual-axis line plot depicting cardiac index (L/min/m², left Y-axis) and cumulative urine output (liters, right Y-axis) over 24 hours from surgical incision. The plot captures hemodynamic performance alongside renal function, showing increasing cardiac output and urine production during the postoperative course in the intensive care unit (ICU).

**Figure 7:**
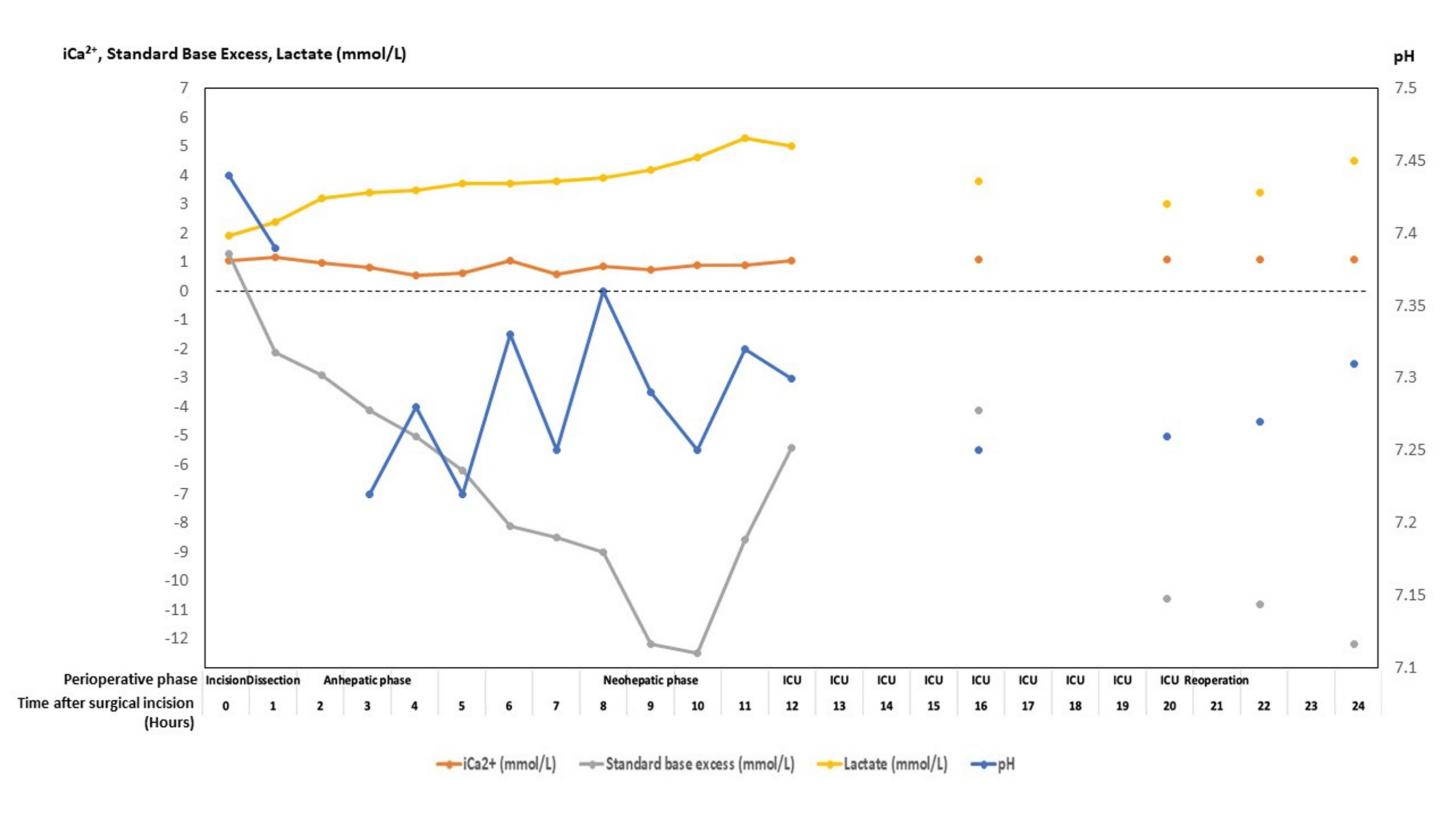
Ionized calcium, standard base excess, lactate, and pH trends over the perioperative and ICU period Line plot showing hourly measurements of ionized calcium (iCa²⁺, mmol/L), standard base excess (mmol/L), lactate (mmol/L), and arterial pH from the start of surgical incision through the intraoperative phases (incision, dissection, anhepatic, neohepatic), followed by the intensive care unit (ICU) stay and reoperation period. This graph illustrates dynamic changes in acid-base balance, calcium homeostasis, and metabolic status critical to patient management during liver transplantation and ICU recovery.

**Figure 8:**
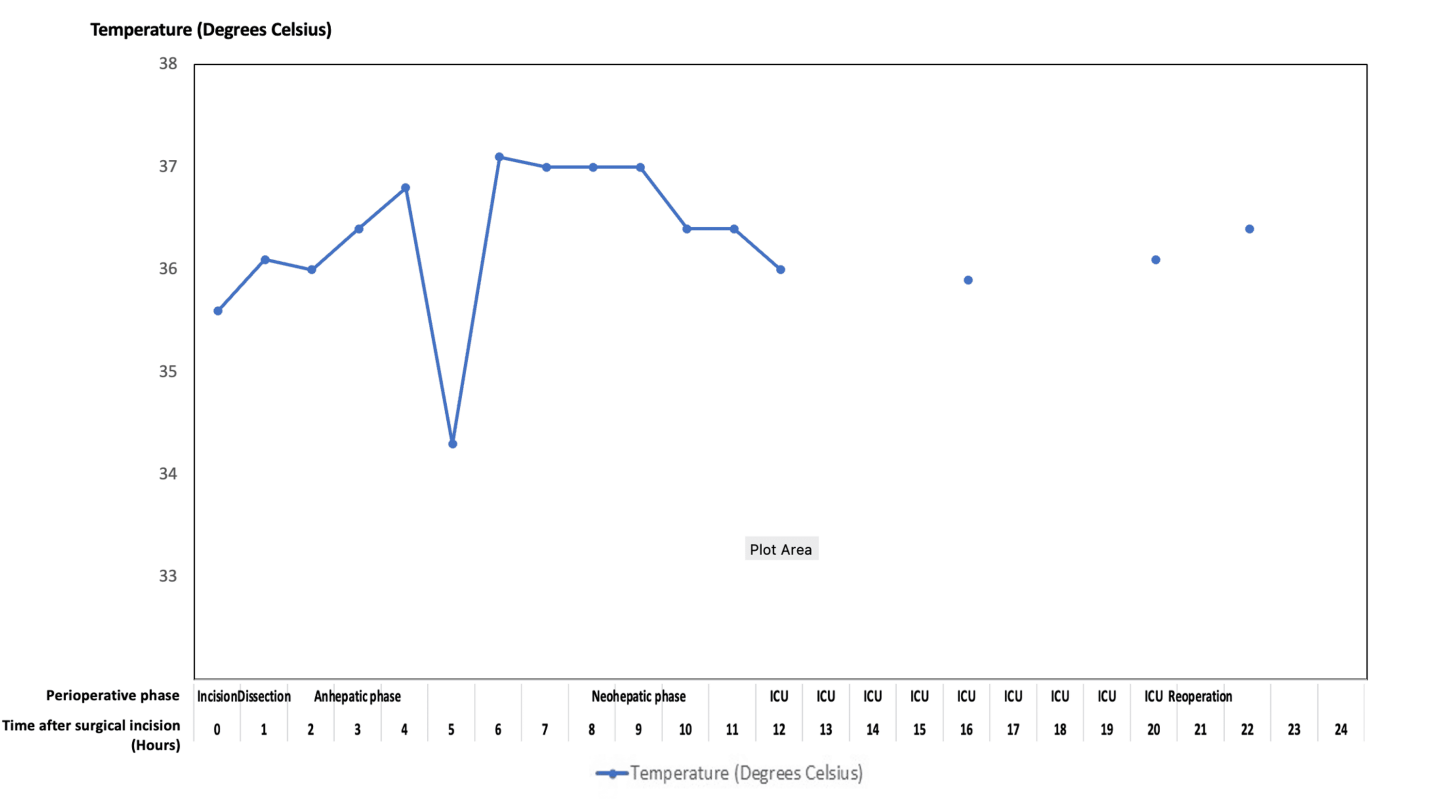
Temperature trend over perioperative and ICU periods Line plot illustrating the patient’s core body temperature (°C) measured hourly from the start of surgical incision through the intraoperative phases (incision, dissection, anhepatic, neohepatic) and into the intensive care unit (ICU) stay and reoperation period. This graph highlights periods of hypothermia and recovery relevant to coagulation and hemodynamic stability during liver transplantation.

Figures 5-8 summarize key physiologic variables during the perioperative and intensive care unit (ICU) periods. Trends in mean arterial pressure (MAP), central venous pressure (CVP), mean pulmonary artery pressure (mPAP), and mixed venous oxygen saturation (SvO₂) (Figure 5) demonstrate fluctuating hemodynamics consistent with vasoplegic shock and volume shifts. Cardiac index and urine output (Figure 6) show progressive improvement post-reperfusion, reflecting gradual stabilization of cardiac function and renal perfusion. Acid-base parameters and blood gases (Figure 7) highlight persistent metabolic acidosis and decreasing ionized calcium during massive transfusion, temporally correlating with worsening hemodynamics and escalating vasopressor requirements. The temperature profile (Figure 8) illustrates intraoperative hypothermic episodes corrected by active warming measures. These data visualizations enhance clinical interpretability by linking physiological derangements to therapeutic interventions. Detailed numerical data are provided in the Appendix.

Postoperatively, the patient was transferred to the ICU. His shock state exhibited a multifactorial aetiology. Initially, he manifested refractory vasoplegic shock, characterized by a high cardiac output and low systemic vascular resistance, which required high-dose infusions of noradrenaline (50 µg/min) and vasopressin (0.04 units/hour). Concurrently, ongoing bleeding and significant blood loss - as evidenced by surgical drain output at a rate of approximately 1 L/hour during the initial two postoperative hours - contributed to a severe component of hemorrhagic shock. Massive transfusion continued in the ICU with administration of an additional 16 PRBC units, four FFP units, two extended-life plasma units, eight pooled platelet units, 20 cryoprecipitate units, 36 µg of desmopressin, and 1 g of tranexamic acid. Additionally, 10 mg of recombinant factor VIIa was administered for refractory coagulopathy. After 4.5 hours, total surgical drain output reached 5 liters, prompting re-laparotomy. Figure 4 depicts the total volume of red blood cells and coagulation factors transfused.

Urgent re-exploration revealed old, clotted blood in the abdomen, patent portal vein and hepatic artery, and a mildly congested liver with watery bile production. A bleeding site near the right adrenal gland was identified; hemostasis was achieved by oversewing the vessel and applying topical hemostatic agents. A 6-French tube was placed into the bile duct as a stent to maintain patency and facilitate postoperative cholangiography. On postoperative day (POD) 4, the patient returned electively to the operating room for primary closure of the bile duct and abdominal wound.

Outcomes

On postoperative day (POD) 8, the patient required retransplantation due to primary graft non-function. The procedure was performed using venovenous bypass with femoral outflow via the portal vein conduit and inflow via the right axillary vein. The bile duct was transected above the anastomosis, and the hepatic artery and portal vein were dissected free. Bypass was initiated, and the caval anastomoses were excised using bicaval clamps. The donor liver was implanted via side-to-side cavocavostomy without complication. Hepatic arterial anastomosis was performed at the gastroduodenal-common hepatic arterial junction. Biliary reconstruction was completed uneventfully, and the abdomen was closed primarily.

The patient’s 51-day ICU stay was complicated by sepsis (source: intra-abdominal), respiratory failure necessitating tracheostomy, bile leak managed with endoscopic retrograde cholangiopancreatography (ERCP) and biliary stenting, portal vein thrombosis treated with therapeutic anticoagulation, and cute kidney injury requiring temporary renal replacement therapy. Following multidisciplinary rehabilitation and sustained medical management, the patient achieved full recovery. He was discharged home five months after the initial transplantation.

At the one-year post-transplant follow-up, the patient demonstrated normalization of graft function, as evidenced by biochemical parameters: bilirubin 19 μmol/L, ALT 18 units/L, AST 32 units/L, albumin 37 g/L, and creatinine 86 μmol/L. All of these values fall within established reference ranges for adults with normal hepatic synthetic function, confirming stable graft performance.

Furthermore, the patient has resumed all prior activities of daily living, indicating successful post-transplant functional recovery. Based on this clinical status, the patient’s Karnofsky performance status is estimated at 90-100%, reflecting the ability to carry on normal activity with minimal or no symptoms of disease.

## Discussion

This challenging case highlights several important learning points. Despite the identification of several patient and surgical factors associated with increased transfusion requirements during LT, consensus on how UMT should be managed in this context remains elusive [6,7]. While a fixed ratio approach to administering blood products is standard care for critical bleeding in trauma settings, such protocols have yet to be established for LT patients [8]. This patient population poses many unique surgical and anesthetic challenges related to coagulopathy, the consequences of massive transfusion, and graft preservation [9]. Therefore, whether it is appropriate to standardize massive transfusion practices in this setting remains a subject of debate [10]. In the presented case, excessive pre-anhepatic bleeding, hypocalcemia, metabolic acidosis, and hypothermia all uniquely contributed to variations in hemostasis during the LT. Therefore, we advocated for a dynamic, goal-directed approach that was tailored to the patient, their laboratory results, and the use of point-of-care VET [11].

In this case, significant surgical bleeding occurred during the pre-anhepatic phase due to the dissection of dense adhesions from the patient’s previous cholecystectomy. By the third hour of surgery, the patient’s hemoglobin had dropped by 3.2 g/L, and noradrenaline requirements had tripled. However, it was difficult to determine the exact relative contribution to the falling hemoglobin level of the surgical bleeding and the dilutional effect of crystalloid administration. Excessive transfusion of blood products and crystalloids likely contributed to dilutional anemia and coagulopathy, a finding well-reported in the literature [12]. 

In contrast, many uncomplicated orthotopic liver transplantations (OLTx) have been safely performed with minimal or no transfusion of blood products, particularly when total blood loss is limited to approximately 2500-3500 mL [13,14]. This highlights the extraordinary nature of our patient’s transfusion volume, totaling over 130 liters, which far exceeds typical reported values. Such massive transfusion cases are exceedingly rare and pose unique management challenges, underscoring the need for individualized, goal-directed therapy in this context.

This case also highlights the precipitous changes in ionised calcium levels during massive transfusion (see Figures 3, 7). Hypocalcemia occurs due to the chelation of calcium and citrate, a compound often contained in blood products. It is a common complication of massive transfusion and leads to increased mortality [12,15]. In addition to this patient’s existing liver impairment, profound hemorrhagic shock likely further impaired the clearance of citrate. Despite ongoing calcium replacement, the patient’s ionized calcium continued to fall to as low as 0.53 mmol/L. Calcium plays a critical role in clotting factor activity, myocardial contraction, and vascular tone [15]. One institution recommends the empirical administration of 2 g of calcium gluconate for every two to four units of PRBC [16].

In the present case, by the fifth hour of surgery, massive transfusion began. Bleeding persisted, with the patient having received a total of 18 units of PRBC while requiring 60 µg/min of noradrenaline. He had received 10 g of calcium chloride at this point, equivalent to 135 mEq of elemental calcium, yet he remained profoundly coagulopathic. Whilst not used in this case, a previous case report has demonstrated the clinical benefits of utilizing continuous QTc monitoring when correcting calcium levels during massive hemorrhage [17]. Implementing such monitoring in our case may have alerted the treating team to the opportunity for more aggressive calcium replacement to help correct both the hemodynamic and coagulopathic effects. Retrospective hemodynamic data showed that the patient’s cardiac index was 2.1 and 2.4 during the critical two-hour period when his ionized calcium levels fell below 1 mmol/L. 

The patient was also severely acidemic, with a pH ranging from 7.22 to 7.28 between operative hours three and five. Metabolic acidosis is commonly associated with chronic liver disease [18] and may occur as a result of massive hemorrhage during the pre-anhepatic or dissection phases, accumulation of lactate and reduced lactate clearance during the anhepatic phase, or prolonged ischemia during the reperfusion phase [4,19]. Biochemically, acidosis exacerbates coagulopathy by impairing the binding of clotting factors to platelets, reducing the activity of certain clotting factors, inhibiting thrombin generation, accelerating fibrinogen degradation, and stimulating fibrinolysis by inducing tissue plasminogen activator (tPA) expression [12,20]. In our case, sodium bicarbonate-containing solution was administered to mitigate acidosis-related coagulopathy; however, its efficacy in improving lactic acidosis outcomes or mortality remains controversial [21]. Alternative strategies, such as intraoperative continuous renal replacement therapy, have been suggested as superior for the correction of acidosis [22,23].

By the fifth hour, the patient’s hemoglobin levels had increased to 164 g/L after 18 PRBC units and 27 L of autologous RBC. The elevated hemoglobin level was confirmed by the central laboratory, excluding an artifact value. Several factors are therefore likely to have contributed to this elevated hemoglobin level, including over-transfusion and transient hemoconcentration due to plasma volume shifts, ongoing bleeding, and the timings of fluid and blood product administration. Whilst these factors may exaggerate the hemoglobin result, leading to a temporary increase in hemoglobin concentration that does not necessarily reflect the true red blood cell mass. Serial hemoglobin levels obtained from arterial blood gas sampling may also be inaccurate in the presence of active bleeding [24]. Further, cognitive overload, which is a well-documented challenge for clinicians in complex and dynamic clinical situations, such as in this case, may have further contributed to a possible over-transfusion of packed RBC [25]. While we did not use continuous non-invasive hemoglobin monitoring devices in this case, their use could help to minimize the effects of these excessive fluctuations in hemoglobin values in the future [26]. 

Further challenges faced in this case included the prolonged duration of the anhepatic phase, which exceeded the six hours. This was attributed to the surgical challenges of locating the thrombosed portal vein and the need for a jump graft. Subsequently, the patient’s temperature dropped to a low of 34.3 degrees Celsius. Hypothermia reduces thrombin generation, inhibits clotting factors, and impairs platelet function [27,28]. Interventions implemented for hypothermia included active warming, using a fluid warmer system, and controlling the ambient operating room temperature. Additionally, the absence of clotting factor production and fibrinogen deficiency during the anhepatic phase further worsened the coagulopathy [29]. 

While the trauma literature has demonstrated the benefits of a fixed 1:1:1 transfusion ratio (i.e., four units RBC: four units FFP: one pooled bag platelets), the unique coagulopathic abnormalities of chronic liver disease and LT may render this approach less desirable [30]. Multiple studies have explored the association between platelet and FFP transfusions in LT, suggesting an inverse relationship between the amount of transfusion and adverse outcomes [31-34]. Some authors suggest that while transfusions are critical for managing coagulopathy during LT, the excessive transfusion of platelets and FFP can paradoxically increase the risk of hepatic artery and portal vein thrombosis, thereby increasing mortality, potentially due to the over-correction of hemostasis or other adverse effects [33,34]. 

**Figure 9:**
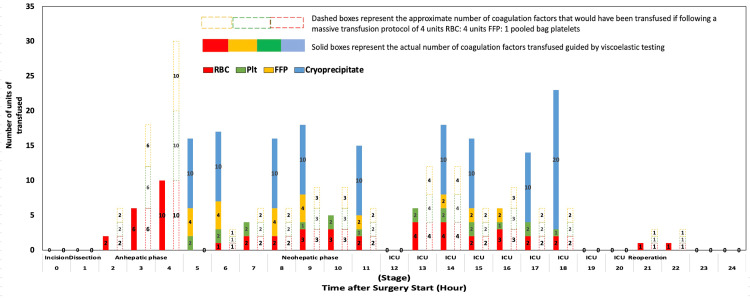
Coagulation products administered guided by viscoelastic testing compared to a theoretical fixed ratio massive transfusion protocol. RBC: red blood cells, Plt: platelets, FFP: fresh frozen plasma, ICU: intensive care unit

Although standard laboratory testing is unreliable in the context of ESLD [35], VET, employed in this case, has been shown to be superior in detecting and monitoring coagulopathy in this population while also guiding the management of these patients during LT [35-38]. Figure 9 demonstrates the coagulation products administered in this case, guided by VET, and compares this to what would have been administered if we employed a massive transfusion protocol using a 1:1:1 fixed ratio approach, i.e., four units RBC: four units FFP: one pooled bag platelets. 

## Conclusions

In summary, over a 24-hour period - including return to theater for surgical hemorrhage control - the patient received a total of 132 L of fluid comprising 50 PRBC units, 20 pooled platelet units, 24 FPP units, and 100 cryoprecipitate units. To our knowledge, this represents one of the most severe cases of intraoperative transfusion and fluid resuscitation documented in the surgical literature. This case emphasizes the multifaceted approach to bleeding management in patients with ESLD and highlights the need for further research into optimal strategies for UMT in this unique clinical context. It also underscores the critical role of monitoring and managing hypocalcemia during ultra-massive transfusion, the challenges and risks associated with over-transfusion and interpretation of hemoglobin levels during rapid transfusion, and the dynamic, complex nature of coagulopathy in ESLD patients undergoing surgery. Management of high-volume intraoperative bleeding during LT via UMT should, rather than utilizing standard, fixed-ratio methods, involve a patient-tailored, goal-directed approach that optimizes all aspects of coagulopathy to achieve optimal patient outcomes.

## Appendices

**Table 2:** Perioperative blood gas and haemodynamic variables.

Time (hours from start of surgery)	0	1	2	3	4	5	6	7	8	9	10	11	12	16	20	22	24
	Incision	Dissection phase		Anhepatic phase						Neohepatic phase			Arrival ICU	4 hours	Reoperation for bleeding	16 hours	24 hours
Blood gas variables																	
pH	7.44	7.39		7.22	7.28	7.22	7.33	7.25	7.36	7.29	7.25	7.32	7.3	7.25	7.26	7.27	7.31
iCa^2+ ^(mmol/L)	1.04	1.18	0.99	0.83	0.53	0.62	1.05	0.58	0.84	0.75	0.91	0.9	1.07	1.1	1.11	1.08	1.09
Standard base excess (mmol/L)	1.3	-2.1	-2.9	-4.1	-5.0	-6.2	-8.1	-8.5	-9.0	-12.2	-12.5	-8.6	-5.4	-4.1	-10.6	-10.8	-12.2
Lactate (mmol/L)	1.9	2.4	3.2	3.4	3.5	3.7	3.7	3.8	3.9	4.2	4.6	5.3	5.0	3.8	3.0	3.4	4.5
Haemodynamic variables																	
Central venous pressures (mmHg)	10	10	5	10	6	13	12	13	14	16	15	17	14	13	15	10	11
Pulmonary artery pressures (mmHg)	27/14	24/15	16/8	16/8	24/11	30/20	34/21	35/18	30/18	44/25	39/18	49/25	32/19	33/22	38/17	40/24	41/20
Cardiac Index (mmHg)	3	3.5	2.1	2.4	2.7	4.1	4.3	3.8	4.6	4.6	5.2	4.9	5.0	4.4	4.3	4.7	4.7
Mean arterial pressure (mmHg)	68	66	77	75	57	69	74	71	66	71	65	60	61	63	65	66	69
Mixed venous oxygen saturations (%)	75	81	82	78	84	76	78	81	69	81	78	78	78	82	81	79	83
Temperature (Degrees Celsius)	35.6	36.1	36	36.4	36.8	34.3	37.1	37	37	37	36.4	36.4	36.0	35.9	36.1	36.4	36.2
Cumulative urine output (mL)	230	240	250	310	450	560	600	660	750	830	860	860	900	980	1100	1200	1210
